# Protection against UVB-Induced Photoaging by *Nypa fruticans* via Inhibition of MAPK/AP-1/MMP-1 Signaling

**DOI:** 10.1155/2020/2905362

**Published:** 2020-06-22

**Authors:** Hee-Jeong Choi, Md Badrul Alam, Mi-Eun Baek, Yoon-Gyung Kwon, Ji-Young Lim, Sang-Han Lee

**Affiliations:** ^1^Department of Food Science And Biotechnology, Graduate School, Kyungpook National University, Daegu 41566, Republic of Korea; ^2^Food and Bio-Industry Research Institute, Inner Beauty/Antiaging Center, Kyungpook National University, Daegu 41566, Republic of Korea; ^3^knu BnC, Daegu 41566, Republic of Korea

## Abstract

Ultraviolet B (UVB) irradiation is major causative factor in skin aging. The aim of the present study was to investigate the protective effect of a 50% ethanol extract from *Nypa fruticans* (NF50E) against UVB-induced skin aging. The results indicated that NF50E exerted potent antioxidant activity (IC_50_ = 17.55 ± 1.63 and 10.78 ± 0.63 *μ*g/mL for DPPH and ABTS-radical scavenging activity, respectively) in a dose-dependent manner. High-performance liquid chromatography revealed that pengxianencin A, protocatechuic acid, catechin, chlorogenic acid, epicatechin, and kaempferol were components of the extract. In addition, the extract exhibited elastase inhibitory activity (IC_50_ = 17.96 ± 0.39 *μ*g/mL). NF50E protected against UVB-induced HaCaT cell death and strongly suppressed UVB-stimulated cellular reactive oxygen species generation without cellular toxicity. Moreover, topical application of NF50E mitigated UVB-induced photoaging lesions including skin erythema and skin thickness in BALB/C mice. NF50E treatment inhibited UVB-induced collagen degradation as well as MMP-1 and IL-1*β* expressions and significantly stimulated SIRT1 expression. Furthermore, the extract treatment markedly suppressed the activation of NF-*κ*B and AP-1 (p-c-Jun) by deactivating the p38 and JNK proteins. Taken together, current data suggest that NF50E exhibits potent antioxidant potential and protection against photoaging by attenuating MMP-1 activity and collagen degradation possibly through the downregulation of MAPK/NF-*κ*B/AP-1 signaling and SIRT1 activation.

## 1. Introduction

The skin protects against pathogens and external damage and acts as a crucial barrier between the internal and external environments of the body. Exposure to chronic ultraviolet (UV) irradiation can lead to adverse pathological effects including skin damage [1]. UV-induced photoaging, which is characterized by modifications of the dermal extracellular matrix (ECM), leads to the development of wrinkles, fragility, laxity, coarseness, impaired wound healing, and increased epidermal thickness [2]. Furthermore, excessive ultraviolet B (UVB) irradiation causes the generation of intracellular reactive oxygen species (ROS). This results in oxidative stress and skin inflammation through the activation of mitogen-activated protein kinase (MAPK) and upregulation of transcription factors, such as activator protein 1 (AP-1) and nuclear factor kappa B (NF-*κ*B) [3, 4]. In addition, UVB-stimulated ROS can enhance the expression of matrix metalloproteinase-1 (MMP-1) in fibroblasts, promoting skin photoaging [5]. MMP-1 degrades collagen type 1, a major ECM component that provides structural support to the skin, and leads to the decomposition of the dermis and skin aging [6]. Therefore, the development of antiaging agents that inhibit UVB-induced ROS generation is essential for suppressing the photoaging process.

SIRT1, a NAD-dependent class III histone deacetylase, plays a vital role in lifespan extension and aging suppression and is regarded as a “longevity protein” [7]. Recent studies have demonstrated that an age-related reduction in SIRT1 levels may be associated with aging biomarkers found in dermal fibroblast cells, which are required for the production of ECM in the skin [8]. A recent study found that SIRT1 could decrease *β*-galactosidase and senescence biomarkers and attenuate the aging of skin lesions [9].

*Nypa fruticans* Wurmb. belongs to the family of *Arecaceae* and is regarded as an “underutilized” plant [10]. *Nypa fruticans* (NF) is predominantly distributed throughout India, Malaysia, Indonesia, and the Philippines and has been traditionally used for the medicinal treatment of conditions such as asthma, leprosy, rheumatism, and pain [10]. NF has been reported to exert various biological activities including antihyperglycemic, antinociceptive, antidiabetes, and antioxidant effects [11, 12]. However, there are no reports regarding the protective effect of NF on photoaging. Accordingly, based on the known effects of NF, this study is aimed at investigating the potential protective effects of NF against UVB-induced skin aging *in vitro* and *in vivo* to develop novel, naturally sourced antiphotoaging agents.

## 2. Materials and Methods

### 2.1. Preparation of Plant Extract

**Figure 1:**
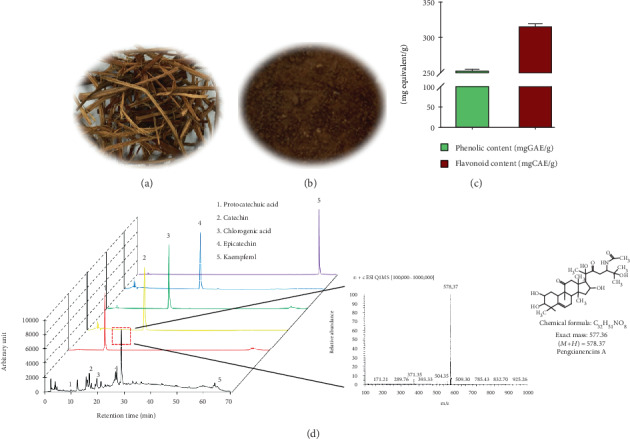
Characteristics of *Nypa fruticans* (NF) and an HPLC chromatogram of the extract. (a) Classical features of *Nypa fruticans* and (b) the powder form is shown. (c) Measurement of total phenolic and flavonoid contents of NF50E. GAE: gallic acid equivalent, CAE: caffeic acid equivalent. (d) Protocatechuic acid (peak 1), catechin (peak 2), chlorogenic acid (peak 3), epicatechin (peak 4), and kaempferol (peak 5) were detected as major components by high-performance liquid chromatography (HPLC) in a 50% ethanolic fraction of *Nypa fruticans* (NF50E). The HPLC chromatogram was recorded at 280 nm along with standard compounds. The dotted box denotes the major peak, which was identified as pengxianencin A by mass spectroscopy analysis (molecular structure of pengxianencin A).

NF was obtained from an online market specializing in agriculture and marine products. NF was dried at 37°C using a dryer (Sanyo convection oven, Osaka, Japan) and ground into a fine powder (Figures 1(a) and 1(b)). Then, a 10-fold volume of ethanol (50%, *v*/*v*) was added to the sample and placed in a shaking incubator for 24 h at 60°C. The 50% ethanolic extract of NF (NF50E) was filtered (Whatman No. 1; Schleicher & Schuell, Keene, NH, USA) and incrassated using a vacuum rotary evaporator (Tokyo Rikakikai Co. Ltd., Tokyo, Japan). Subsequently, the sample was lyophilized using a freeze dryer (Il-shin Biobase, Goyang, Korea) and stored at 4°C. The extract was dissolved in dimethyl sulfoxide (DMSO) or distilled water for experimental use.

### 2.2. High-Performance Liquid Chromatography (HPLC) Analysis and Mass Spectroscopy

The phytochemical characteristics of NF50E were identified by HPLC using a Shimadzu Prominence Auto Sampler (SIL-20A) HPLC system (Shimadzu, Kyoto, Japan) equipped with an SPD-M20A diode array detector (PDA) and LC solution 1.22 SP1 software. Protocatechuic acid, chlorogenic acid, catechin, epicatechin, and kaempferol were used as standard compounds. Reverse-phase chromatographic analysis was performed using a Phenomenex C18 column (4.6 mm × 250 mm) packed with 5 *μ*m diameter particles. A stepwise gradient of solvent A to B was used (A: 2% acetic acid and B: 50% acetonitrile (CAN) in 0.5% acetic acid). The flow rate was 0.8 mL/min, and the injection volume was 10 *μ*L. A Q-Exactive™ Quadrupole-Orbitrap™ mass spectrometer (Thermo Fisher Scientific Inc., Rockford, IL, USA) was used to perform the mass experiments. The settings of the IT mass spectrometer were as follows: ESI voltage +4 kV, nebulization with N2 at 1.7 bar, dry gas flow 7 L/min, gas temperature 310°C, skimmer 1 voltage +12.4, collision energy set to 1 V, and ramped within 40%–200% of this value. The ion number accumulated within the trap was set to 10,000, and the maximum accumulation time was 200 ms. To determine the key chemical sdiagnostic product ions over the full range, the product ion spectrum was recorded in the targeted mode for the mass range m/z 50–1500.

### 2.3. Antioxidant Assays

NF50E was selected for measurement of total phenolic content (TPC) and total flavonoid content (TFC). The analysis of TPC was done by using the Folin Ciocalteu reagent [13]. Folin Ciocalteu reagent was added to a distilled water-diluted sample at a 1 : 10 ratio, and 11 mL of the resulting solution was stored at 25°C. 2 mL of Na_2_CO_3_ 20% solution was added, incubated for 1 h, and the absorbance of the mixture was measured at 595 nm. The TFC was measured according to a previously reported method [14]. Potassium acetate solution (0.1 ml of 0.1% (*v*/*v*)) and 0.1 mL of 10% (*w*/*v*) AlCl_3_ were mixed with 2.8 mL of distilled water, and a 0.5 mL sample was diluted with 1.5 mL of methanol, and the two solutions were mixed. The mixtures were kept at room temperature for 30 min, and the absorbance was measured at 405 nm. The results of TPC and TFC were expressed as mg gallic acid-equivalents (GAE) or catechin-equivalents (CAE) per 100 mg of extract, respectively, as described elsewhere [13].

2,2-Diphenyl-1-picrylhydrazyl (DPPH) and 2,20-azino-bis 3-ethylbenzothiazoline-6-sulphonic acid (ABTS) radical scavenging assays, ferric reducing antioxidant power (FRAP) assay, and cupric reducing antioxidant capacity (CUPRAC) assay were conducted to evaluate the hydrogen and electron-donating capacity of NF50E. We also confirmed the cell-free antioxidant activity of NF50E as described previously [13].

### 2.4. Elastase Inhibition Assay

The elastase inhibitory activity of NF50E was assessed according to a previously reported method with minor modifications [14]. Briefly, the reaction mixture contained 0.1 M Tris-HCl buffer (pH 8.0), 0.78 mM N-succinyl-Ala-Ala-Ala-p-nitroanilide (Sigma-Aldrich, St. Louis, MO, USA), and 0.04 unit/mL elastase (45124; Sigma-Aldrich, St. Louis, MO, USA) with or without 2 *μ*L of NF50E in 96-well plates (SPL Life Sciences Co., Ltd., Pocheon, Korea). The absorbance at 405 nm was measured 25 times at 1 min intervals using a UV spectrophotometer at 37°C (Victor3; PerkinElmer, Waltham, MA, USA). Epigallocatechin gallate (EGCG) was used as a positive control.

### 2.5. Cell Culture and Cell Viability Assay

HaCaT-immortalized human keratinocytes were purchased from AddexBio Technologies (San Diego, CA, USA). The cells were maintained in DMEM supplemented with 10% fetal bovine serum and 1% penicillin-streptomycin at 37°C in a 5% CO_2_ humidified atmosphere. A 3-(4,5-dimethyl-2-thiazolyl)-2,5-diphenyl-2H-tetrazolium bromide (MTT) assay was performed to assess cell viability according to a previously described method [15]. Briefly, HaCaT cells were cultured at a density of 1 × 10^5^ cells/mL in 96-well plates and incubated at 37°C for 24 h in a CO_2_ incubator. When the cells were 80–90% confluent, various concentrations of NF50E (1, 3, 10, 30, and 100 *μ*g/mL) were added followed by further incubation for 24 h. The media was changed with the MTT (5 mg/mL in phosphate-buffered saline; PBS) solution and incubated for an additional 1 h. Subsequently, the MTT solution was removed and 100 *μ*L of DMSO was added to dissolve the formazan crystals. The optical density was measured using a microplate reader at 595 nm (Victor3; PerkinElmer, Waltham, MA, USA).

For UVB irradiation experiments, HaCaT cells (1 × 10^5^ cells/mL) were seeded in 96-well plates and incubated at 37°C for 24 h in a CO_2_ incubator. The cells were then treated with different concentrations of NF50E for an additional 24 h. The media were discarded, 100 *μ*L of PBS was added, and the cells were exposed to UVB (30 mJ/cm^2^) radiation using a UV lamp (Bio-Link Crosslinker; Vilber Lourmat, Cedex, France). The overall average dose of UVB radiation exposure was set at 8.01 mJ/cm^2^/d according to a previous report [16]. In this experiment, we used UVB radiation at 30 mJ/cm^2^, which is equivalent to about 4 days of sun exposure. The media was then replaced with fresh media and predetermined concentrations of NF50E (1~100 *μ*g/mL) were added. After 24 h of further incubation, cell viability was measured by MTT assay.

### 2.6. Measurement of Intracellular ROS

The redox-sensitive dye H_2_DCFDA was used to measure the production of intracellular ROS. HaCaT cells were harvested (1 × 10^5^ cells/mL) in 96-well black clear-bottom plates for 24 h. Different concentrations of NF50E were added to the cells along with 25 *μ*M DCFH-DA for 1 h. After washing with 100 *μ*L of PBS, the cells were exposed to UVB (30 mJ/cm^2^) radiation. After 30 min, the fluorescence intensity was measured using a microplate reader (Victor3; PerkinElmer, Waltham, MA, USA) at excitation and emission wavelengths of 485 and 535 nm, respectively.

### 2.7. UVB-Induced Experimental Mouse Model

Balb/c mice (20–22 g) aged 7 weeks were obtained from Samtako Korea (Osan, Korea). The mice were housed in a temperature- and humidity-controlled room (22 ± 1°C, 55 ± 1%) under a 12 h dark/light cycle with free access to commercial diet and water. The study was approved by the Committee on Laboratory Animal Ethics (KNU 2017-0029), Kyungpook National University (Daegu, Korea). The mice were divided into five groups of five mice each as follows: UV(−)+Vehicle (G1), UV(+)+Vehicle (G2), UV(+)+EGCG (10 mg/mL) (G3), UV(+)+NF50E at a concentration of 10 mg/mL (G4), and UV(+)+NF50E at a concentration of 50 mg/mL (G5). The dorsal skin of each mouse was shaved using a hair trimmer and hair removal cream. Each mouse was treated with 150 *μ*L of sample solution, followed by exposure to UVB radiation. The UV intensity was gradually increased from 1 MED to 4 MED using the experimental schedule described in Supplementary Figure S1. In the preliminary experiment, UVB radiation of 75 to 300 mJ/cm^2^ for 25 days was sufficient to induce acute skin inflammation in the mice. Saline and 1,3-butylene glycol at a 3 : 7 volume ratio were used as vehicles. Skin appearance was evaluated by visual observation and photographs were taken using a Nikon camera (D5100; Nikon, Tokyo, Japan). The skin thickness and level of erythema were measured using a digimatic thickness gauge (Code No. 547-315; Mitutoyo, Kanagawa, Japan) and colorimeter (CR-400; Minolta, Tokyo, Japan), respectively, to measure the Δa∗ value and skin erythema index [17].

### 2.8. Histochemical and Immunohistochemical Analyses

After the sacrifice of all mice, tissue samples were obtained from the dorsal skin. The dorsal skin tissues were immobilized in 10% formaldehyde solution in PBS for 24 h and embedded in paraffin. Slices were cut at 5 *μ*m thickness, and the sections were deparaffinized prior to soaking in acetone and washing with PBS. The slides were treated with 3% hydrogen peroxide in methanol to block peroxidase activity, and epitope retrieval was conducted. Subsequently, the samples were incubated with 10% normal goat serum for 1 h. SIRT1 (ab166821; Abcam), MMP-1 (ab137332; Abcam), and IL-1*β* (ab9722; Abcam) were used as primary antibodies and incubated with the sections overnight. Hematoxylin and eosin (H&E) staining and Masson's trichrome staining were performed to examine the skin thickness and collagen content in the dermis, respectively. Stained slides were visualized by microscopy (ECLIPSE TE2000-U; Nikon, Tokyo, Japan).

### 2.9. RNA Isolation and Reverse Transcription-Polymerase Chain Reaction (RT-PCR)

Total RNA from the mouse dorsal skin samples was isolated using TRIzol reagent (Life Technologies; Carlsbad, CA, USA) according to the protocol described elsewhere [13]. Equal amounts of RNA (2 *μ*g) were used as a template for the synthesis of cDNA using the RT-&GO Master Mix (MP Biomedicals, Santa Ana, CA, USA). The amplified products were electrophoresed on 1% agarose gels, visualized with ethidium bromide, and visualized using Image Lab software (ChemiDoc). GAPDH was used for normalization.

### 2.10. Western Blot Analysis

Homogenized skin tissues were lysed in buffer containing protease and a phosphatase inhibitor. The proteins were quantified using the Bradford protein method [18]. The proteins (50 *μ*g) were separated by 10% sodium dodecyl sulfate-polyacrylamide gel electrophoresis (SDS-PAGE) and transferred to nitrocellulose membranes (Whatman, Dassel, Germany). The membranes were blocked with 5% skim milk or bovine serum albumin for 1 h and washed with Tris-buffered saline including Tween-20 (TBST) for 1 h at 15 min intervals. The membranes were incubated with primary antibodies against MMP-1 (ab137332; Abcam), SIRT1 (ab166821; Abcam), ERK 1/2 (BS 6472, Bioworld Technology, Inc., Nanjing, China), phospho-ERK 1/2 (sc-7383, Santa Cruz Biotechnology, Inc.), JNK (sc-7345, Santa Cruz Biotechnology, Inc.), phospho-JNK (BS 4322, Bioworld Technology, Inc.), p38 (BS3567, Bioworld Technology, Inc.), phospho-p38 (sc-166182, Santa Cruz Biotechnology, Inc.), NF-*κ*B (BS1254, Bioworld Technology, Inc.), and phospho-c-Jun (BS4050, Bioworld Technology, Inc.) at 4°C overnight. After rinsing, the membranes were incubated with anti-rabbit IgG-horseradish peroxidase (HRP) (Bethyl Laboratories, Montgomery, TX, USA), anti-mouse IgG-HRP (Bethyl Laboratories, Montgomery, TX, USA), and anti-goat IgG-HRP (Bethyl Laboratories, Montgomery, TX, USA) as secondary antibodies for 2 h. Proteins were detected using an ECL solution system (ChemiDoc™ XRS+; Bio-Rad).

### 2.11. Statistical Analysis

The results are presented as the mean ± standard deviation (SD) using triplicate values. Statistical differences between the mean values were determined by Tukey's one-way ANOVA test using IBM SPSS Statistics software (Armonk, NY, USA). Differences were considered significant at *p* < 0.05.

## 3. Results

### 3.1. HPLC Analysis of a 50% Ethanolic Extract of NF

As shown in Figure 1(c), NF50E contained several polyphenolics and flavonoid compounds. To confirm which polyphenolic compounds were present in NF50E, HPLC analysis was performed. Protocatechuic acid, catechin, chlorogenic acid, epicatechin, and kaempferol were detected at the following retention times: protocatechuic acid (12.378 min), catechin (19.691 min), chlorogenic acid (21.241 min), epicatechin (26.218 min), and kaempferol (64.184 min) (Figure 1(d)). In addition, mass spectroscopy (positive ion mode) revealed that the major peak at 28.88 min was a cucurbitane triterpenoid, pengxianencins A, m/z 578.37 (M+H)^+^, calculated by the molecular formula C_32_H_51_NO_8_ [19].

### 3.2. Effect of NF50E on Antioxidant Activity

**Figure 2:**
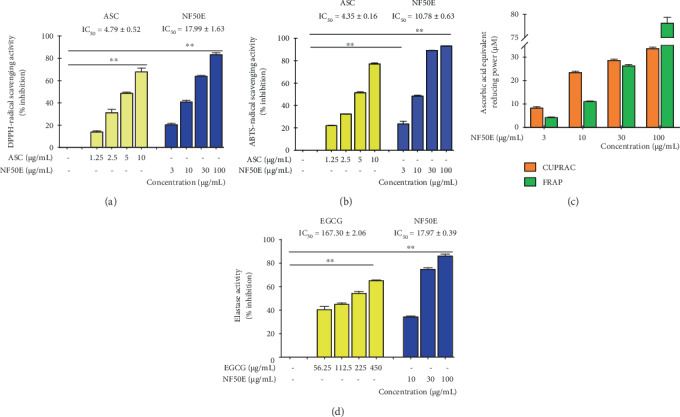
Antioxidant and elastase inhibitory effects of NF50E. (a) DPPH-radical scavenging assay, (b) ABTS-radical scavenging assay, (c) cupric reducing antioxidant capacity (CUPRAC) assay and ferric reducing antioxidant power (FRAP) assay, (d) elastase inhibition assay were performed with various concentrations of NF50E. Ascorbic acid (ASC) or (−)-epigallocatechin gallate (EGCG) was used as a positive control. The results are shown as means ± SD performed in triplicate (^∗∗^*p* < 0.05).

In order to investigate the antioxidant capacity of NF50E, various *in vitro* assays such as DPPH and ABTS-radical scavenging assays, FRAP assay, and CUPRAC assay were performed. In the DPPH and ABTS-radical scavenging assays, NF50E exhibited significant concentration-dependent radical scavenging activity with IC_50_ values of 17.99 ± 1.63 and 10.78 ± 0.63 *μ*g/mL, respectively (Figures 2(a) and 2(b)). In addition, ascorbic acid, a positive control, showed more potent DPPH and ABTS-radical scavenging activity with IC_50_ values of 4.79 ± 0.52 and 4.35 ± 0.16 *μ*g/mL, respectively. The FRAP and CUPRAC values obtained for NF50E increased with increasing concentration (Figure 2(c)). These results demonstrate the strong antioxidant activity of NF50E.

### 3.3. Effect of NF50E on Elastase Inhibitory Activity

Elastase breaks down elastin and elastic fibers, and the inhibition of elastase activity could prevent the degradation of elastin, one of the major components of the ECM. The results revealed that the elastase inhibitory activity of NF50E was the highest compared with that of a DW extract of *Nypa fruticans* (NFD) and a 100% EtOH extract of *Nypa fruticans* (NFE), with an IC_50_ value of 16.88 ± 1.16 *μ*g/mL (Figure 2(d), and Supplementary S6). Based on these results, NF50E was selected for further analysis.

### 3.4. Effect of NF50E on HaCaT Cell Viability

**Figure 3:**
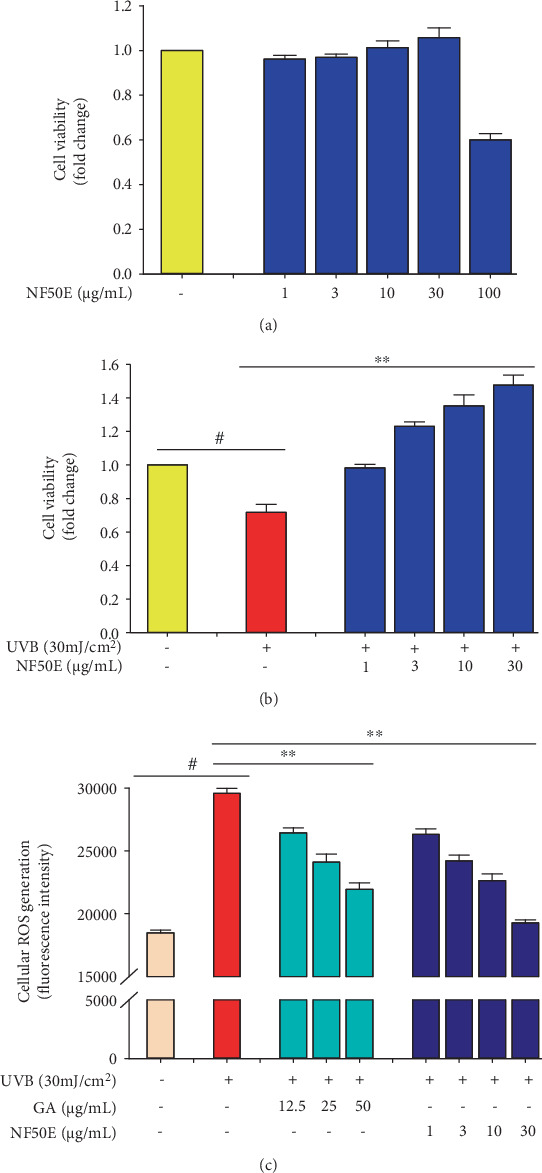
Cell viability and inhibition of reactive oxygen species (ROS) generation by NF50E in HaCaT cells. Cytotoxicity of NF50E without (a) or with (b) UVB (30 mJ/cm^2^) were calculated by 3-(4,5-dimethylthiazol-2-yl)-2,5-diphenyltetrazolium bromide (MTT) assay. The experiments represent the mean ± SD. ^#^*p* < 0.05 versus the nonirradiated group, ^∗∗^*p* < 0.05 versus the UV-irradiated group. (c) Intracellular reactive oxygen species (ROS) levels were measured according as described in Materials and Methods. Gallic acid was used as a positive control. Values represent the mean ± SD. ^#^*p* < 0.05 compared with the non-UV-treated group, ^∗∗^*p* < 0.05 versus the UV-treated group. GA: gallic acid.

Before the start of the cell experiment, an MTT assay was performed to confirm the toxicity of any extract or single molecule. To assess the toxicity effect of NF50E on HaCaT cells, various concentrations of NF50E (1, 3, 10, 30, and 100 *μ*g/mL) were evaluated in an MTT assay. As shown in Figure 3(a), NF50E had no cytotoxic effects up to 30 *μ*g/mL. Thus, 1, 3, 10, and 30 *μ*g/mL of NF50E were used for further studies.

Next, to evaluate whether NF50E could protect against cell death from UVB irradiation, an MTT assay was performed. As shown in Figure 3(b), exposure to UVB (30 mJ/cm^2^) for 24 h induced cell death (23.76 ± 7.71%) compared with the nonirradiated group. Interestingly, NF50E treatment protected against UVB-induced cell death in a concentration-dependent manner up to 1.4-fold.

### 3.5. Effect of NF50E on UVB-Induced ROS Production

Increasing evidence has indicated that ROS is one of the major causes of UVB-stimulated cellular senescence by damaging DNA strands and/or altering DNA bases [20]. To examine whether NF50E treatment could suppress UVB-induced cellular ROS production, a DCFDA-ROS detection assay was performed. As expected, UVB irradiation (30 mJ/cm^2^) significantly increased cellular ROS production compared with production in the nonirradiated group (Figure 3(c)). However, NF50E treatment suppressed UVB-stimulated cellular ROS formation in a dose-dependent manner.

### 3.6. Effect of NF50E on Cutaneous Changes in a UVB-Induced Mouse Model

**Figure 4:**
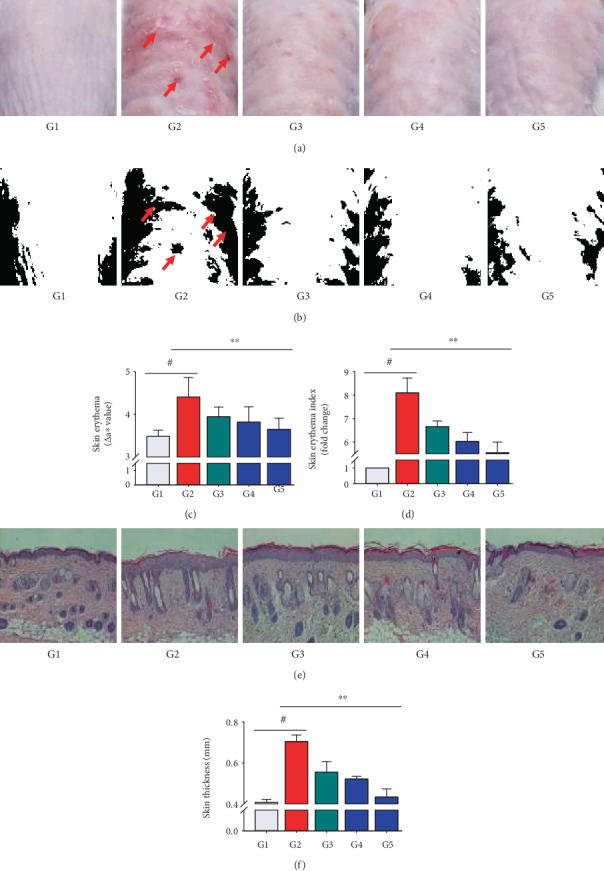
Changes in skin lesions of UVB-treated Balb/c mice by NF50E. The experiment group was divided as follows: UV(−)+Vehicle (G1), UV(+)+Vehicle (G2), UV(+)+EGCG (10 mg/mL) (G3), UV(+)+NF50E at a concentration of 10 mg/mL (G4), and UV(+) + NF50E at a concentration of 50 mg/mL (G5). (a) Photograph of representative skin surface of the dorsal skin of BALB/C mice (*n* = 5) after UVB treatment. (b) Skin erythema on the dorsal skin (red color) was processed using Image J software. (c) A colorimeter was used to calculate skin erythema and expressed as a Δa∗ value. (d) Skin erythema index was calculated using Image J software. (e, f) Skin thickness was measured by hematoxylin and eosin (H&E) staining (epidermis) and a digimatic thickness gauge (dorsal thickness), respectively. Values are expressed as means ± SD (*n* = 5), ^#^*p* < 0.05 versus the non-UV-irradiated group; ^∗∗^*p* < 0.05 versus the UV-irradiated group.

To investigate the antiphotoaging potential of NF50E *in vivo*, the dorsal skin of mice was exposed to UVB as described in Materials and Methods (Supplementary Figure S1). As shown in Figure 4(a), the dorsal skin of the UVB-irradiated group was wrinkled, rough, dry, flaky, and reddish compared with that of the nonirradiated group; however, topical application of NF50E protected against UVB-induced lesions. Moreover, skin erythema was induced on the dorsal skin of the UVB-irradiated group (2^nd^ images in Figures 4(a) and 4(b)) compared with the nonirradiated vehicle-treated group (1^st^ images in Figures 4(a) and 4(b)), and NF50E treatment mitigated UVB-stimulated skin erythema (4^th^ and 5^th^ images in Figures 4(a) and 4(b), and Figures 4(c)-4(f)).

As shown in Figures 4(e), H&E staining demonstrated that UVB irradiation led to an increase in epidermal skin thickness compared with the nonirradiated group, whereas NF50E treatment reduced UVB-induced epidermal thickening. As expected, compared with nonirradiated mice, UVB-irradiated mice exhibited a thicker dorsal skin, and NF50E treatment significantly restored the skin thickness to near-normal levels (Figure 4(f)).

### 3.7. Effect of NF50E on SIRT1 Expression in the UVB-Induced Mouse Model

**Figure 5:**
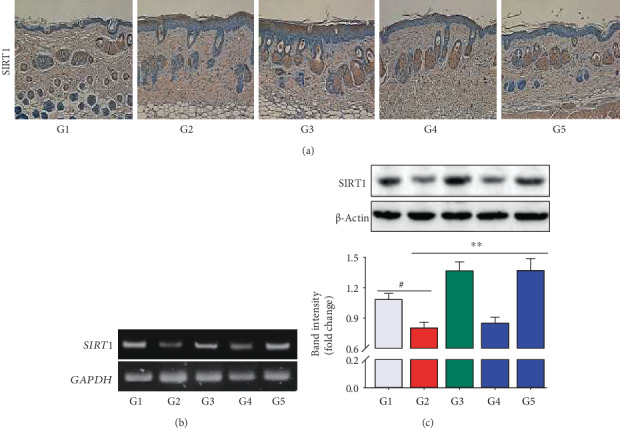
Effects of NF50E on SIRT 1 expression in UVB-stimulated mice. The dorsal skin of mice was collected and fixed with formaldehyde and embedded in paraffin as described in Materials and Methods. (a) Immunohistochemical staining with anti-SIRT1, (b) mRNA expression of SIRT1, and (c) immunoblotting analysis was performed. UV(−)+Vehicle (G1), UV(+)+Vehicle (G2), UV(+)+EGCG (10 mg/mL) (G3), UV(+)+NF50E at a concentration of 10 mg/mL (G4), and UV(+)+NF50E at a concentration of 50 mg/mL (G5). Values are expressed as means ± SD (*n* = 5), ^#^*p* < 0.05 versus the non-UV-irradiated group; ^∗∗^*p* < 0.05 versus the UV-irradiated group.

In Figure 5, immunohistochemical analysis revealed that UVB exposure decreased SIRT1 secretion in the dermis (brown color) along with the destroyed skin layer. NF50E treatment reversed this trend, but low expression of SIRT1 in the EGCG treatment group was evident (Figure 5(a)). Furthermore, RT-PCR and immunoblotting analyses revealed similar results (Figures 5(b) and 5(c)), demonstrating that NF50E stimulated SIRT1 secretion, thereby protecting skin from the photoaging process.

### 3.8. Effect of NF50E on Skin Aging Biomarker Expression in a UVB-Induced Mouse Model

**Figure 6:**
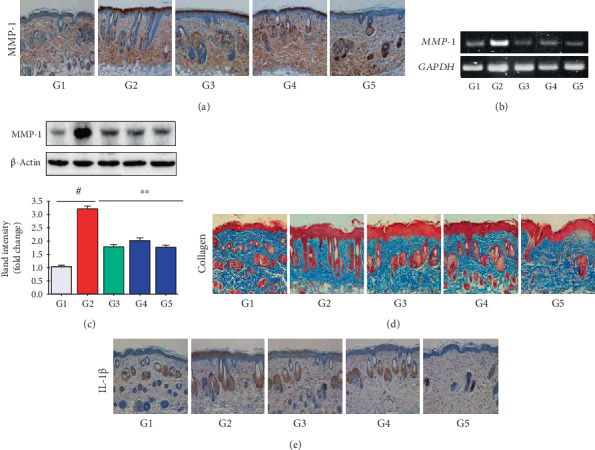
Effects of NF50E on skin aging-related biomarkers. (a-c) Expression of matrix metalloproteinases- (MMP-) 1 was confirmed using immunohistochemical staining (a), reverse transcription-polymerase chain reaction (RT-PCR) (b), and western blot analysis (c). (d) Collagen density in dorsal skin of mice was measured using Masson's trichrome staining. (E) Interleukin-1 beta (IL-1*β*) was analyzed by immunohistochemistry. UV(−)+Vehicle (G1), UV(+)+Vehicle (G2), UV(+)+EGCG (10 mg/mL) (G3), UV(+)+NF50E at a concentration of 10 mg/mL (G4), and UV(+)+NF50E at a concentration of 50 mg/mL (G5). Values are expressed as means ± SD (*n* = 5), ^#^*p* < 0.05 versus the non-UV-irradiated group; ^∗∗^*p* < 0.05 versus the UV-irradiated group.

Matrix metalloproteinases (MMPs) can degrade various ECM containing proteins such as collagen, fibronectin, elastin, and proteoglycans and contribute to photoaging [21]. In this study, RT-PCR revealed that the expression of MMP-1, MMP-8, and MMP-13 was significantly upregulated in the UVB-irradiated group compared with the non-irradiated group, and NF50E and EGCG treatment prevented this effect (Figure 6(b) and Supplementary Figure S8). Furthermore, both immunohistochemistry and immunoblot analyses revealed that UVB exposure upregulated MMP-1 expression in the epidermis (brown color), and NF50E and EGCG treatment reversed this trend (Figures 6(a) and 6(c)).

Masson's trichrome staining demonstrated that the collagen content in the dermis of the UVB-irradiated group was reduced (blue stain) compared with that of the nonirradiated group; however, treatment with NF50E abrogated the UVB-induced reduction of collagen content in the dermis (Figure 6(d)). As expected, the mRNA expression of COL1A1 was also decreased in the UVB-induced group and NF50E treatment reversed this effect (Supplementary Figure S8).

**Figure 7:**
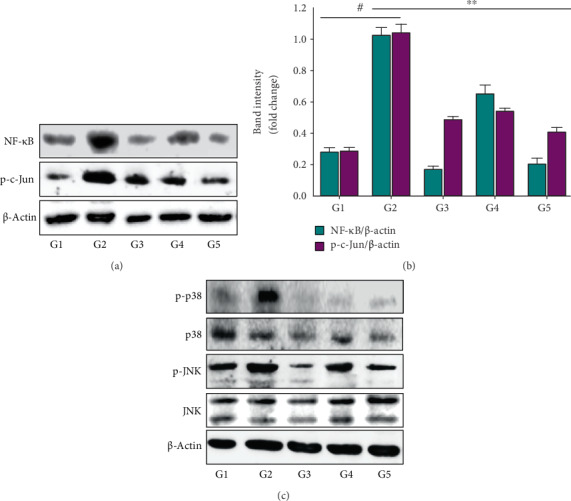
Involvement of nuclear factor kappa B (NF-*κ*B)/mitogen-activated protein kinase (MAPK) and AP-1 signaling pathway by NF50E. (a) Immunoblotting analysis of NF-*κ*B and p-c-Jun. (b) Relative band intensity of the expressions of NF-*κ*B and p-c-Jun were quantified by Image lab software. UV(−)+Vehicle (G1), UV(+)+Vehicle (G2), UV(+)+EGCG (10 mg/mL) (G3), UV(+)+NF50E at a concentration of 10 mg/mL (G4), and UV(+)+NF50E at a concentration of 50 mg/mL (G5). ^#^*p* < 0.05 versus the non-UV-irradiated group; ^∗∗^*p* < 0.05 versus the UV-irradiated group. (c) Effects of NF50E on MAPK which include p38 and JNK signaling pathways were confirmed by western blot analysis.

Active interleukin-1 (IL-1) is found in epidermal keratinocytes, and its expression is enhanced by UVB irradiation, resulting in inflammation [22]. In this study, immunohistochemical assay revealed that UVB irradiation enhanced IL-1*β* expression in the epidermis (brown color), which was suppressed by treatment with NF50E and EGCG (Figure 6(e)). Notably, transcriptional factors including NF-*κ*B and AP-1 play a crucial role not only in regulating MMPs and IL-1*β* but also in maintaining the ECM composition [23]. Since NF50E modulated the expression of MMPs and IL-1*β*, we next investigated whether NF50E could regulate NF-*κ*B and AP-1. Immunoblotting assays demonstrated that both NF-*κ*B (p65) and AP-1 (p-c-Jun) were markedly increased by UVB exposure (Figure 7(a); upper layer and lower layer, respectively); however, NF50E treatment considerably reduced this increase (Figures 7(a) and 7(b)).

### 3.9. Effects of NF50E on the Phosphorylation of MAPK Proteins

We investigated the pathway through which NF50E exerts its antiphotoaging effects. Generally, UVB-augmented ROS production leads to the activation of MAPK proteins including ERK, p38, and JNK. MAPK induced NF-*κ*B and AP-1, consequently enhancing the expression of MMPs and leading to a decrease in collagen and other ECM components in aged skin tissues [23]. To investigate the effects of NF50E on UVB-induced photoaging, the phosphorylation of MAPKs was assessed. The phosphorylation of p38 and JNK was significantly increased in UVB-irradiated cells compared with nonirradiated cells. Treatment with NF50E inhibited the phosphorylation of p38 and JNK (Figure 7(c)), but NF50E did not inhibit the phosphorylation of ERK1/2 (Supplementary Figure S9). These results indicate that the suppression of UVB-stimulated p38 and JNK phosphorylation by NF50E may be required for the attenuation of NF-*κ*B and AP-1 in HaCaT cells.

## 4. Discussion

In this study, we investigated the mechanisms of antiphotoaging by a *Nypa fruticans* extract. In the course of the screening process for potent antiaging biomolecules from food sources, we found that a 50% EtOH extract of *Nypa fruticans* (NF50E) contained various polyphenolics, including protocatechuic acid, catechin, chlorogenic acid, epicatechin, kaempferol, and a cucurbitane triterpenoid, known as pengxianencin A (Figure 1(d)). Among them, protocatechuic acid exhibited not only antiskin aging effects by collagen synthesis and MMP-1 inhibition *in vitro* but also antiwrinkle effects *in vivo* [24]. Protocatechuic acid may be found in various natural sources. In this study, we are the first to identify protocatechuic acid in a *Nypa fruticans* extract, suggesting that the plant extract may possess unique antiaging compounds, which were predicted based on a literature search. These polyphenolics may induce the biosynthesis of elastin, collagen, and other skin matrix proteins, suggesting that they are deeply associated with the inhibition of certain enzymes or with induction of the MMPs during the aging/antiaging process in the skin [25, 26]. Using mass spectroscopy, we identified the main peak of the extract, an alkaloid of the cucurbitane triterpenoid family, known as pengxianencin A (MW = 578.37). This substance was originally discovered in *Hemsleya penxianensis* tubers, and its function was assumed to be involved in self-defense from environmental insects and pathogens [27]. Based on this data, we confirmed that this substance is the main component of the antiaging activity by evaluating its activity *in vitro* and *in vivo*. As shown in Figure 2(d), we knew that NF50E exhibited a potent elastase inhibitory effect. In addition, HaCaT keratinocytes were used to explore the relationship between skin cell senescence and the protective effects of NF50E against UV exposure. There was no toxicity up to a concentration of 30 *μ*g/mL of NF50E, and the extract improved HaCaT cell viability, which was decreased by UVB irradiation (Figures 3(a) and 3(b)). We concluded that the polyphenolic compounds protected cell viability and exhibited an antiaging effect. Thus, as we predicted, the components of NF50E decreased UVB-induced ROS generation and photoaging effects by increasing antioxidant activity *in vitro* and *in vivo*.

Skin represents a protective barrier between internal organs and the environment and the appearance of photo-aged skin is characterized by wrinkles, sagging, erythema, and thickness due to the degradation of ECM proteins [28]. In this study, the topical application of NF50E mitigated the adverse effects on murine dorsal skin (Figures 4(a)-4(d)). Matsumura et al. reported that skin becomes thicker as protection from UV-induced damage when subjected to UV exposure [29]. We discovered from our animal data that skin thickening was attenuated in the NF50E-treated group compared with the UV-irradiated group (Figures 4(e) and 4(f); compare 2^nd^ to 1^st^ and 5^th^ images). At the histological level, it is known that chronically sun-exposed human skin suffers damage to the collagenous extracellular matrix that comprises the skin connective tissue and reduced levels of collagen and elastin [30, 31], as shown in the UV-irradiated G2 group. Because collagen and elastin contribute to the strength and resiliency of the skin, and their degradation from UV-induced aging can result in an aged appearance [32], it is prudent to (i) protect collagen and elastin integrity for skin matrix stability, (ii) promote matrix biosynthesis such as collagen and elastin in the skin, and (iii) inhibit degradation-related enzyme activities in the skin environment.

To further evaluate the mechanism of NF50E, we monitored the expression of antiaging biomarkers after NF50E treatment of HaCaT cells. Because Masson's trichrome staining revealed that NF50E abrogated the UV-induced reduction of collagen density in the dermis (Figure 6(d)), these results strongly suggested that the extract exerted multiple functions against UVB-induced skin damage, resulting in ROS reduction, ECM degradation, and a decrease in collagen and elastin content. Therefore, to investigate the role of molecular signaling pathways attenuated by NF50E, we evaluated the MAPKs and transcription factors. Immunohistochemistry results showed that NF50E enhanced SIRT1 expression while suppressing MMP-1 and IL-1*β* expressions. MMPs are known as calcium-dependent zinc-containing endopeptidases that regulate various physiological processes including apoptosis, inflammation, wound healing, and aging [33, 34]. Activated MMPs lead to the degradation and synthesis inhibition of the ECM and collagen in connective tissues, thereby triggering photoaging [35]. Among the 28 different MMP family members, MMP-1, MMP-8, and MMP-13, which are known as collagenases, recognize substrates through a hemopexin-like domain and can degrade fibrillar collagen [21]. UVB significantly enhanced the mRNA expression level of MMP-1, MMP-8, and MMP-13, whereas NF50E decreased the UVB-stimulated expression of these genes in a dose-dependent manner (Figure 6(b) and Supplementary Figure S8).

**Figure 8:**
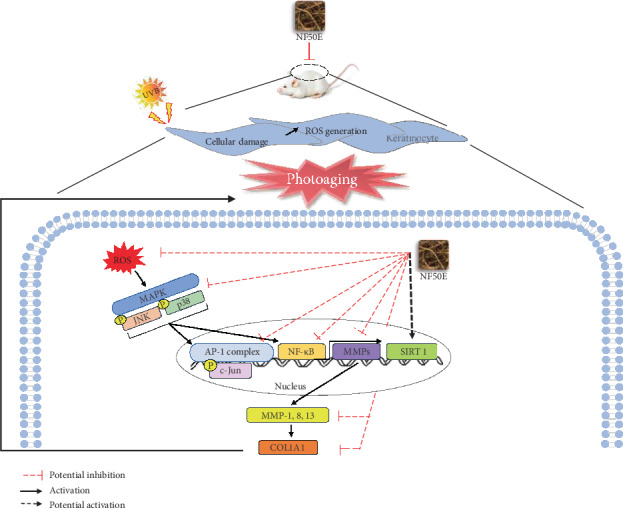
A proposed mechanism for the effects of NF50E on UVB-induced skin aging. NF50E protected photoaging through MAPK-mediated NF-*κ*B signaling, followed by MMP-1 downregulation and SIRT-1 upregulation. Red dotted T bars: potential inhibition; straight arrows: activation; dotted arrows: potential activation,

SIRT 1, a longevity protein with type III histone deacetylase activity, is a member of the sirtuin family and has an important role in cell survival and longevity during cellular senescence [36]. Thus, modulating SIRT1 pathways represents a strategy of suppressing cellular senescence and skin aging [32]. In this study, we provide evidence suggesting that SIRT1 plays a protective role in a UV-induced mouse model. The reduction of the SIRT1 and COL1A1 genes following UVB irradiation was prevented by NF50E treatment (Figure 5 and Supplementary Figure S8). It is now well-documented that MMP-1 is a key enzyme that degrades connective tissues resulting in photoaging [37]. We already confirmed that the protein level of SIRT1 was increased, whereas MMP-1 was decreased by NF50E (Figures 5(c) and 6(c)). There are no decisive reports, however, on the relationship of these two proteins, which may be closely regulated by downstream signaling pathways and transcription factors [38]. A major effector of the MAP kinase pathway is transcription factor AP-1 which consists of the Jun and Fos family proteins. In addition, nuclear factor kappa B (NF-*κ*B) is known to be activated by UV irradiation in skin keratinocytes and increases the expression of MMP-1 in the dermis. Thus, the regulation of NF-*κ*B signaling represents a method of preventing UV-mediated cutaneous alterations or skin photoaging [29, 39]. As expected, after exposure to UVB, the phosphorylation of MAPKs (p38 and JNK) was induced. However, treatment with NF50E markedly suppressed the activation of MAPKs along with the downregulation of NF-*κ*B and AP-1 signaling (Figure 7). Possible mechanisms for the effects of NF50E against skin photoaging are presented in Figure 8. The results in this study demonstrate that NF50E exerts a protective effect against UVB-induced skin aging through the inhibition of MAPKs *in vitro* and *in vivo.* It has been documented that SIRT1 closely interacts with c-Jun [40]. Therefore, SIRT1 controls MMP-1 transcription through downregulation of AP-1 and NF-*κ*B transcription factors, resulting in protein expression by decreased MMP-1 and increased SIRT1 in order to decrease wrinkling.

Nipa (*Nypa fruticans*) was originally cultivated near seashores and swamp areas. Therefore, we surmised that the plant's growth environment may not be easy to replicate in other areas. This may impact the optimal conditions required for producing the active compounds needed for investigation [41]. Small amounts of salt such as sodium chloride are used in the development of skin washes and for removing debris from products, so the active compounds of the plant should be characterized so the ingredients can be developed for the purpose of anti-skin aging [42]. Presently, we cannot determine exactly which compounds (ingredients) are exert activity in the skin. Interestingly, we identified pengxianencin A as a component of the extract, and its precise mechanism on antiaging and crosstalk between MMPs and SIRT1 proteins will provide insight into its role as an active ingredient in the extract.

## 5. Conclusions

In conclusion, the present study showed that NF50E could effectively protect the skin from UVB-induced photoaging. NF50E protected HaCaT cell against UVB radiation by suppressing UVB-induced cellular ROS generation. In *in vivo* assays, photo-aged skin lesions such as erythema and skin thickness were attenuated by NF50E. In addition, NF50E upregulated the expression of SIRT1 and inhibited MMP-1 activity and downregulated NF-*κ*B and AP-1 signaling via phosphorylation of p38 and JNK proteins. Collectively, these findings indicate that NF50E may be used as a natural biomolecule for the development of anti-photoaging foods or skincare products.

## Data Availability

The data used to support the findings of this study are available from the corresponding author upon request.

## Supplementary Materials

Supplementary Materials Figure S1: experimental schedule of UV irradiation intensity and time. 75 to 300 mJ/cm^2^ was the range of UV irradiation; Supplementary Figure S2: total phenolic and flavonoid contents of a DW extract from *Nypa fruticans* (NFD) and a 100% EtOH extract from Nypa fruticans (NFE). Supplementary Figure S3: DPPH-radical scavenging activity of NFD and NFE. Supplementary Figure S4: effects of ABTS-radical inhibition activity by NFD and NFE. Supplementary Figure S5: reducing antioxidant capacity of NFD and NFE. Supplementary Figure S6: elastase inhibition activity of NFD and NFE. Supplementary Figure S7: cell viability of NFD and NFE using an MTT assay. Supplementary Figure S8: effect of NF50E on mRNA expression of Col1a1, MMP-8, and MMP-13 were done by RT-PCR analysis. Supplementary Figure S9: phosphorylation of ERK by NF50E was performed by western blot analysis.
