# Supplementary material for: Protection against UVB-Induced Photoaging by Nypa fruticans via Inhibition of MAPK/AP-1/MMP-1 Signaling
Source: Oxid Med Cell Longev. 2020 Jun 22;2020:2905362. doi: 10.1155/2020/2905362 (PMC7330638; doi:10.1155/2020/2905362)
Supplement: Supplementary Materials — Figure S1: experimental schedule of UV irradiation intensity and time. 75 to 300 mJ/cm2 was the range of UV irradiation; Supplementary Figure S2: total phenolic and flavonoid contents of a DW extract from Nypa fruticans (NFD) and a 100% EtOH extract from Nypa fruticans (NFE). Supplementary Figure S3: DPPH-radical scavenging activity of NFD and NFE. Supplementary Figure S4: effects of ABTS-radical inhibition activity by NFD and NFE. Supplementary Figure S5: reducing antioxidant capacity of NFD and NFE. Supplementary Figure S6: elastase inhibition activity of NFD and NFE. Supplementary Figure S7: cell viability of NFD and NFE using an MTT assay. Supplementary Figure S8: effect of NF50E on mRNA expression of Col1a1, MMP-8, and MMP-13 were done by RT-PCR analysis. Supplementary Figure S9: phosphorylation of ERK by NF50E was performed by western blot analysis. [file 2905362.f1.pptx]

## Slide 1
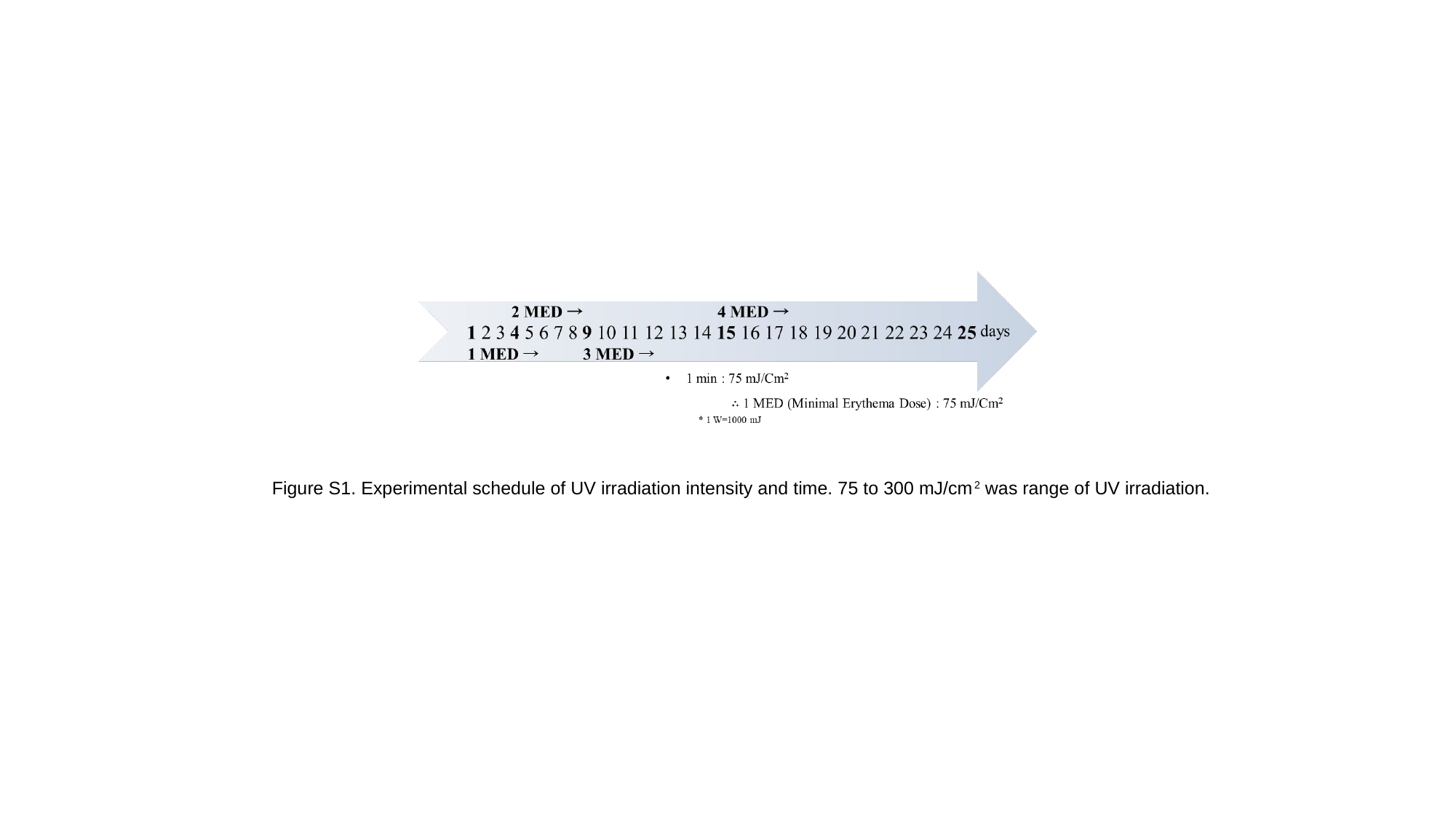

Figure S1. Experimental schedule of UV irradiation intensity and time. 75 to 300 mJ/cm2 was range of UV irradiation.

## Slide 2
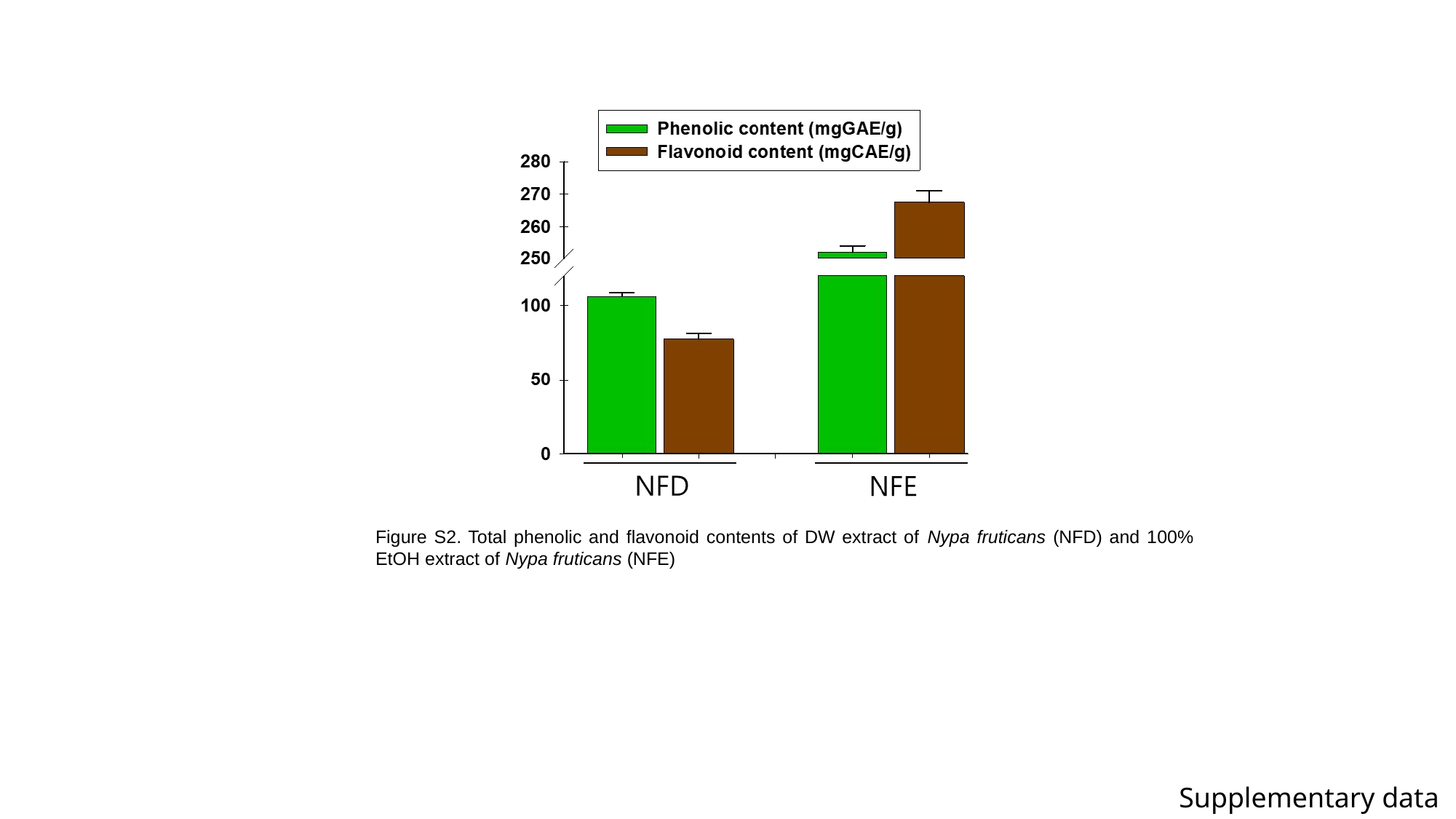

Figure S2. Total phenolic and flavonoid contents of DW extract of Nypa fruticans (NFD) and 100% EtOH extract of Nypa fruticans (NFE)
Supplementary data

## Slide 3
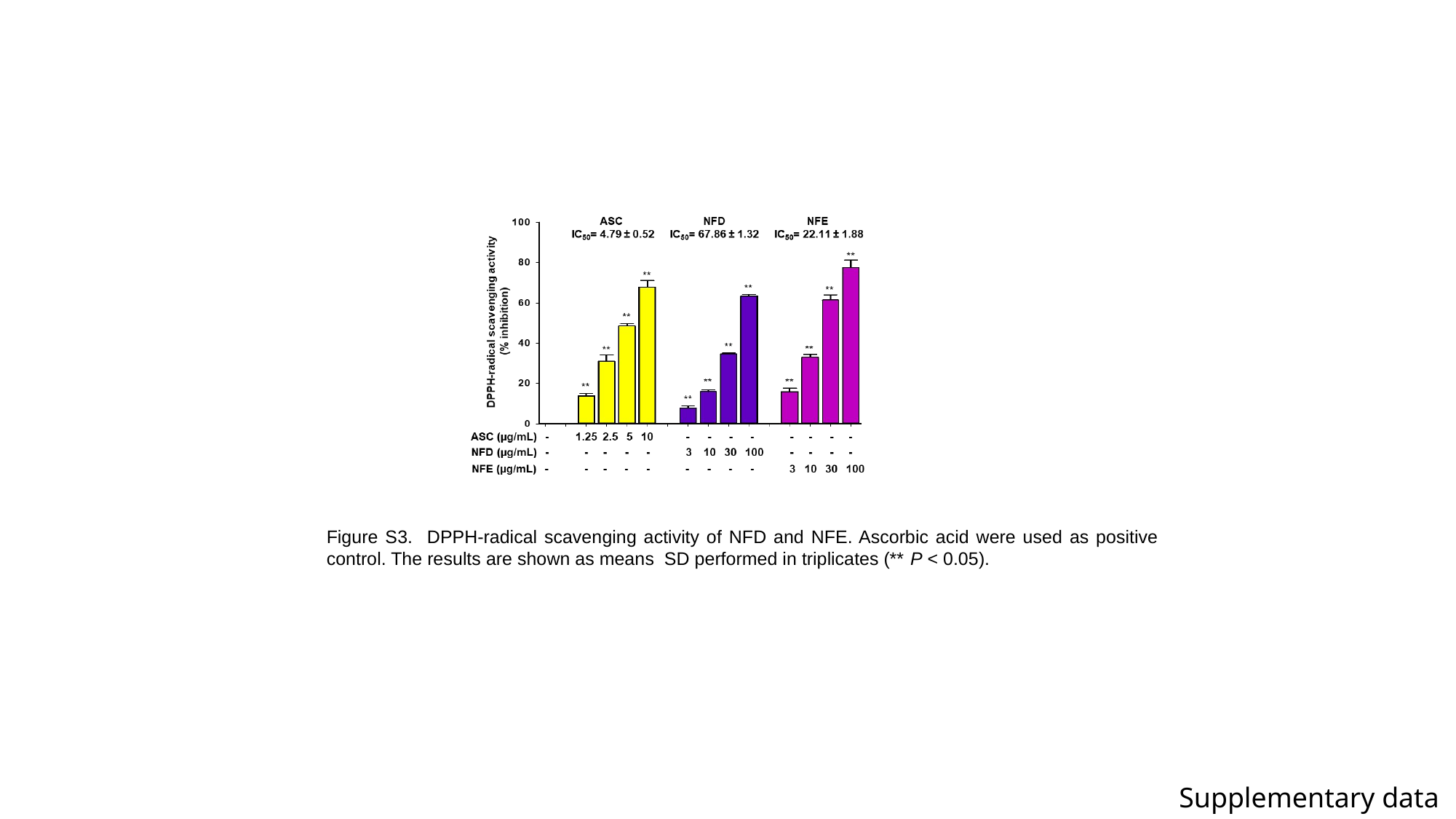

Supplementary data

## Slide 4
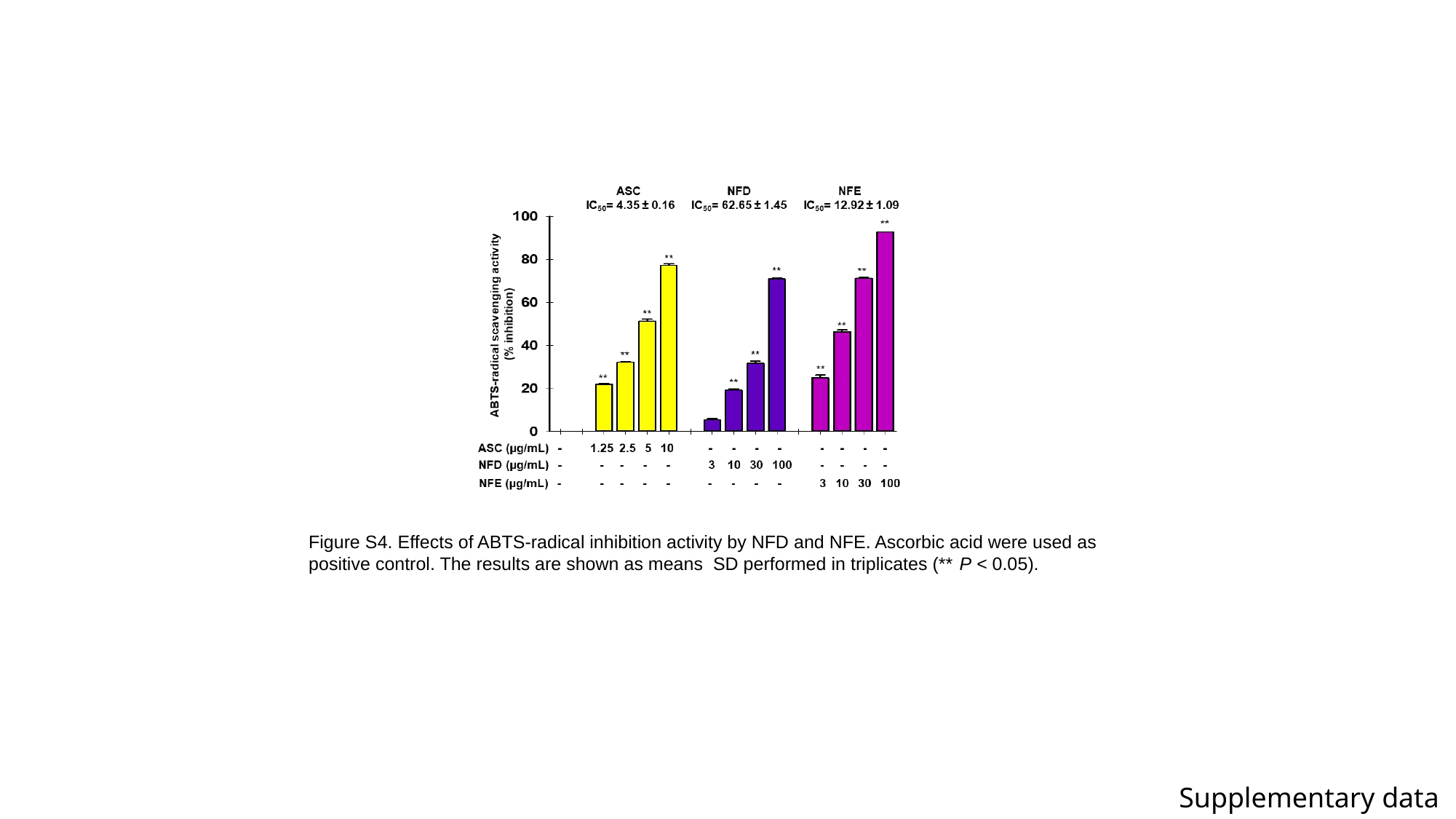

Supplementary data

## Slide 5
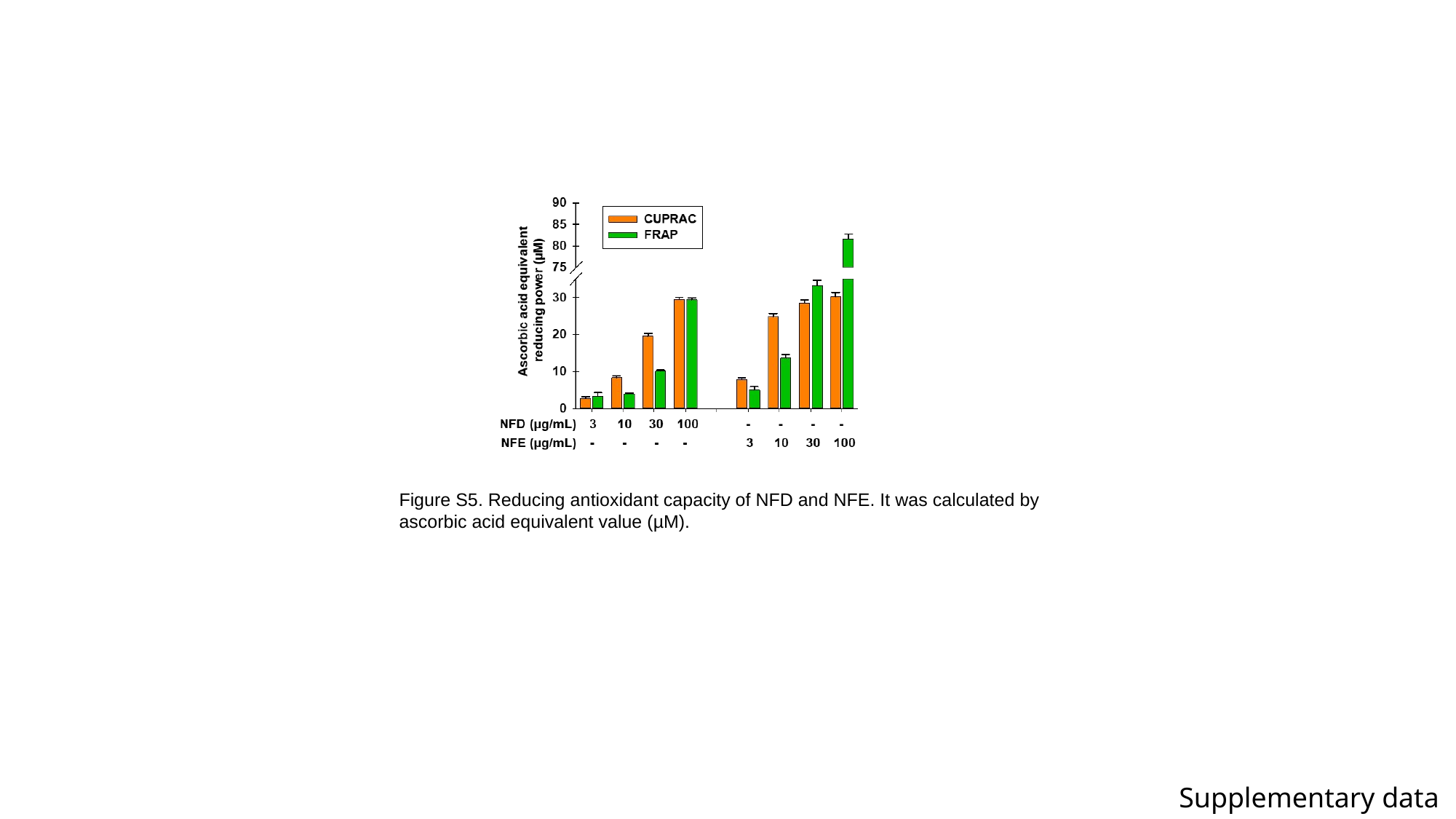

Figure S5. Reducing antioxidant capacity of NFD and NFE. It was calculated by ascorbic acid equivalent value (µM).
Supplementary data

## Slide 6
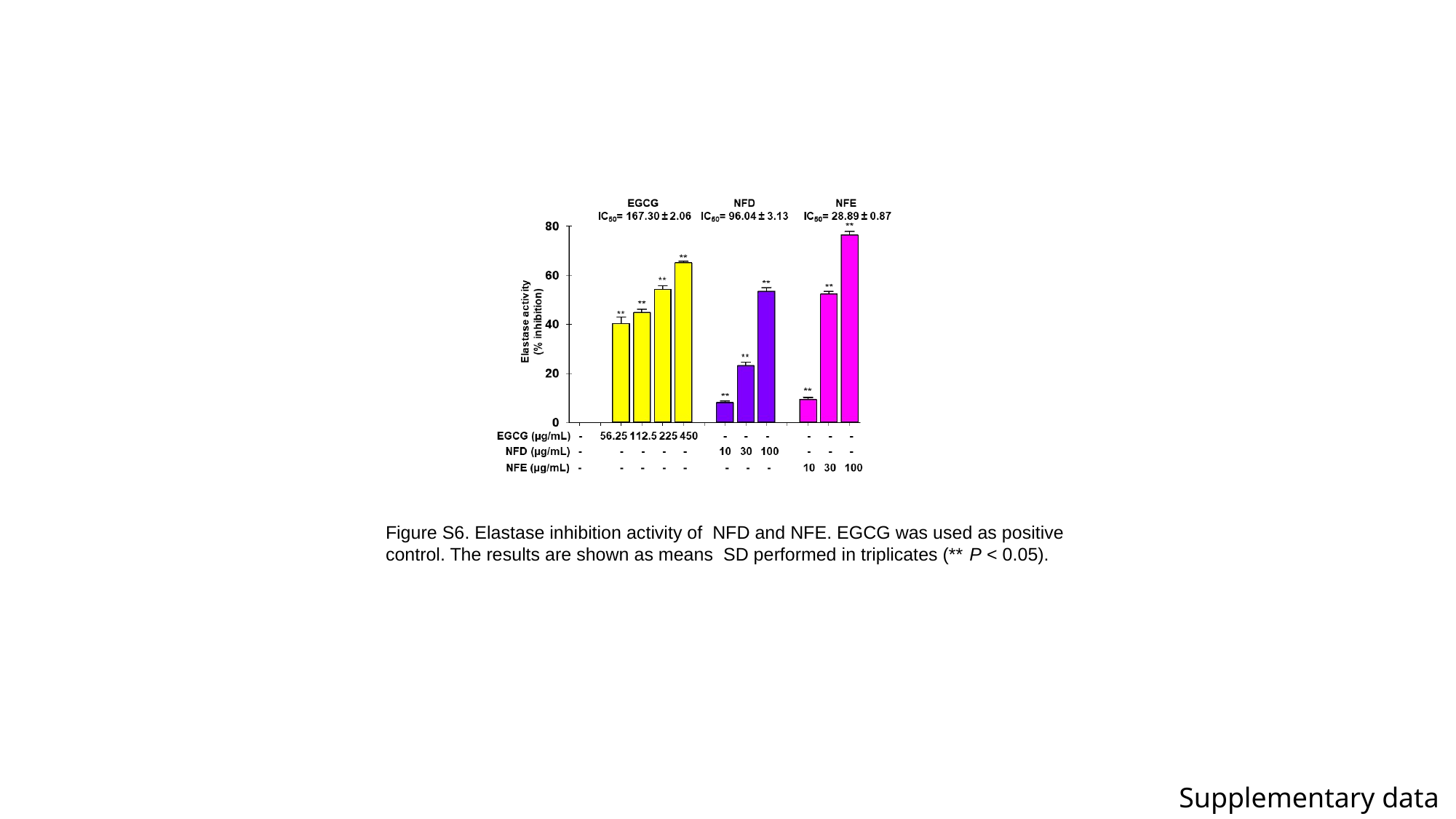

Supplementary data

## Slide 7
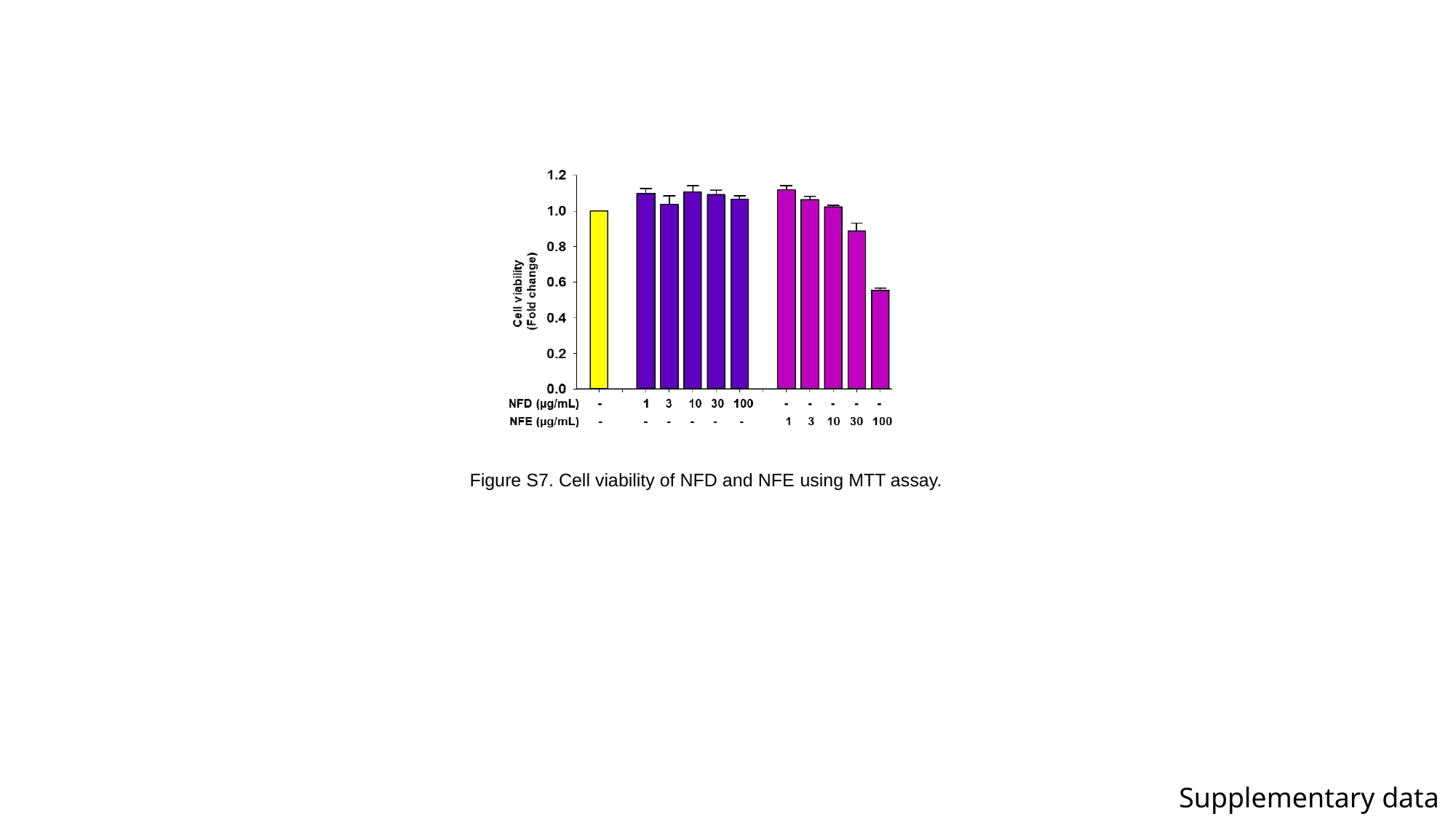

Figure S7. Cell viability of NFD and NFE using MTT assay.
Supplementary data

## Slide 8
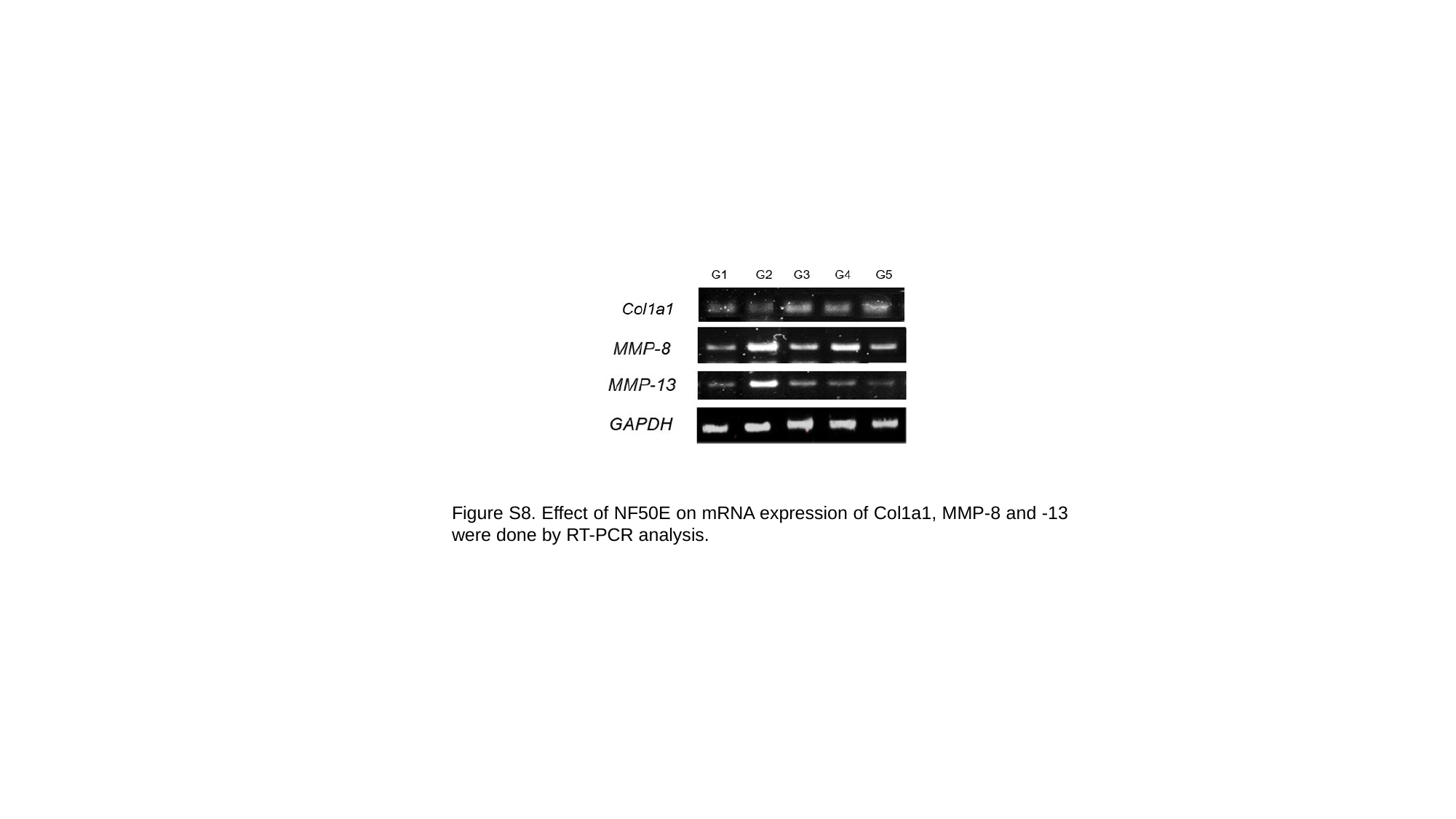

Figure S8. Effect of NF50E on mRNA expression of Col1a1, MMP-8 and -13 were done by RT-PCR analysis.

## Slide 9
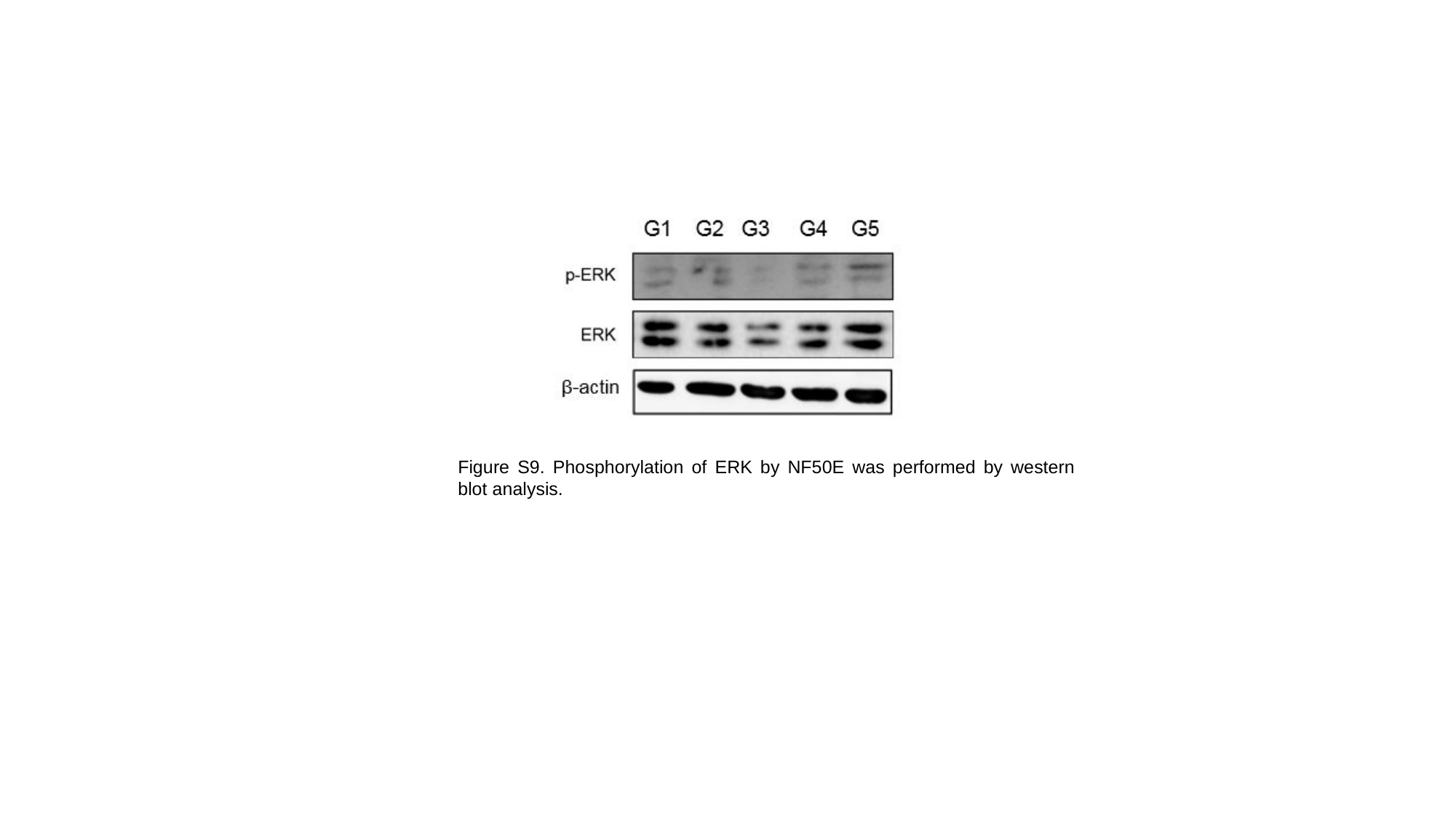

Figure S9. Phosphorylation of ERK by NF50E was performed by western blot analysis.
